# Circulating Cytokines Could Not Be Good Prognostic Biomarkers in a Mouse Model of Amyotrophic Lateral Sclerosis

**DOI:** 10.3389/fimmu.2019.00801

**Published:** 2019-04-12

**Authors:** Laura Moreno-Martínez, Miriam de la Torre, Janne M. Toivonen, Pilar Zaragoza, Alberto García-Redondo, Ana Cristina Calvo, Rosario Osta

**Affiliations:** ^1^LAGENBIO, Faculty of Veterinary-IIS, IA2-CITA, CIBERNED, University of Zaragoza, Zaragoza, Spain; ^2^ALS Unit, Neurology Department, CIBERER U-723, Health Research Institute, Madrid, Spain

**Keywords:** cytokines, biomarkers, SOD1G93A mice, plasma, amyotrophic lateral sclerosis

## Abstract

**Background:** There is growing evidence of the role of inflammation in Amyotrophic Lateral Sclerosis (ALS) during the last decade. Although the origin of ALS remains unknown, multiple potential inflammatory biomarkers have been described in ALS patients and murine models of this disease to explain the progressive motor neuron loss and muscle atrophy. However, the results remain controversial. To shed light on this issue, we aimed to identify novel biomarkers of inflammation that can influence disease progression and survival in serial blood samples from transgenic SOD1G93A mice, a model of ALS.

**Methods:** A cytokine array assay was performed to analyze protein expression of 97 cytokines in plasma samples from wildtype controls and transgenic SOD1G93A mice at asymptomatic stage. Subsequently, serial plasma samples were obtained from SOD1G93A mice at early symptomatic, symptomatic and terminal stages to monitor cytokine levels during disease progression through immunoassays. Comparisons of means of quantifiable cytokines between short-and long-lived mice were analyzed by unrelated *t*-test or Mann-Whitney U-test. Relationships between cytokines levels and survival time were assessed using Pearson's correlation analysis and Kaplan-Meier analysis.

**Results:** A total of 16 cytokines (6Ckine, ALK-1, CD30 L, eotaxin-1, galectin-1, GITR, IL-2, IL-6, IL-10, IL-13, IL-17B R, MIP-1α, MIP-3β, RANKL, TROY, and VEGF-D) were found dysregulated in transgenic SOD1G93A mice at asymptomatic stage compared with age-matched controls. Immunoassays of serial samples revealed positive expression of ALK-1, GITR and IL-17B R at P60 and P90 in mice with shorter survival. In addition, eotaxin-1 and galectin-1 levels were significantly increased at terminal stage in SOD1G93A mice that showed shorter survival time. Finally, levels of eotaxin-1, galectin-1, IL-2, IL-6, MIP-1α, and TROY at P90 or endpoint negatively correlated with the longevity of transgenic mice.

**Conclusions:** We demonstrated in the SOD1G93A model of ALS that increased levels of several cytokines were associated with a shorter lifespan. However, their role as prognostic biomarkers is unclear as their expression was very variable depending on both the disease stage and the subject. Nevertheless, cytokines may be potential therapeutic targets.

## Introduction

Amyotrophic Lateral Sclerosis (ALS) is one of the most common rare diseases of unknown origin that leads to progressive motor neuron degeneration and muscle denervation (1). In particular, it has been described that either distal axonopathy or neuromuscular junction damage precedes motor neuron loss (2). The known mutations that produce the typical adult ALS phenotype are, in order of frequency, the hexanucleotide repeat expansion in C9ORF72, mutations related to the copper/zinc superoxide-dismutase-1 gene (SOD1), Tar DNA-binding protein gene (TARDBP) and DNA/RNA-binding protein FUS (fused in sarcoma) (3, 4). Only 5–10% of ALS cases are familiar, whereas the rest of ALS cases are sporadic, most of them with unknown cause.

The immune system has emerged as one of the key players linked to the development of neurodegeneration, such as in ALS (5–10). The immune system seems to have a dual role, polarizing its functional phenotype toward an inflammatory M1 phenotype or toward an anti-inflammatory M2 phenotype, depending on the particular neurodegenerative environment and by disease stage (7, 11). Although the exact and sequential processes by which the immune system can influence the course of the disease in ALS remains unclear, many studies have suggested immune-related biomarkers can help to predict the progression of the disease (5, 11–13). For example, reactive microglia and astrocytes adjacent to degenerating motor neurons secrete signal molecules to blood, and these may play a key role in the propagation of the disease (12, 14, 15). In this sense, the possibility of studying blood as a matrix to identify of immunologic signals can lead to a better understanding of the disease and to the discovery of novel predictive biomarkers of disease progression.

Expression of several cytokines has been recently described altered in blood of ALS patients. Some studies have pointed out that interleukin (IL)-2 and IL-6 levels together with other interleukins remained increased toward terminal stages in blood samples from ALS patients (13, 16). However, these results are not consistent with some other studies. For instance, IL-6 levels were found to decrease along disease progression in definite ALS patients in a 6-month period, whereas in the same patients IL-2 showed the opposite trend (11). This suggests dynamic alterations in cytokine levels may occur depending on disease stage or the patients themselves. Accordingly, a recent meta-analysis indicated that alterations in some cytokines were inconsistent between published studies (17). These contradictory results may reflect the unavoidable heterogeneity found in ALS patients, which could possibly be mitigated using an animal model maintained under controlled environmental conditions. In this sense, the SOD1G93A mouse model of ALS, has already demonstrated its potential in the discovery of molecular markers that are conserved between human patients (18–20). Regarding immunity, a recent meta-analysis indicated that IL-6 and some other cytokine levels are increased in transgenic SOD1G93A mice (18); however, analysis of cytokines serially monitored along disease progression has not been reported in SOD1G93A mice to date.

Under this complex scenario, we have focused our work in studying levels of inflammatory markers in serial plasma samples from transgenic SOD1G93A mice to identify those that could influence disease progression and survival of mutant animals. The identification of potential prognostic biomarkers involved in inflammation in this model could enhance the successful translation of the results to ALS patients. In addition, this study may contribute to shed light on whether or not cytokines could be reliable prognostic biomarkers for ALS or maybe they could be better considered as potential therapeutic targets.

## Materials and Methods

### Animals

B6SJL-Tg SOD1G93A mice (stock number 002726) were purchased from The Jackson Laboratory (Bar Harbor, ME, USA). Experimental hemizygous transgenic mice were obtained by mating hemizygous SOD1G93A males with C57BL/6J x SJL/J F1 hybrid females (B6SJLF1) purchased from Janvier Labs (Saint-Berthevin Cedex, France). The offspring were identified by PCR assay as described in The Jackson Laboratory protocol. The mice were housed at the animal facilities in Centro de Investigación Biomédica de Aragón under a standard light:dark (12:12) cycle. Food and water were provided *ad libitum*. The humane endpoint for these mice is defined as the loss of righting reflex as shown by a failure to right after laying the mouse on its side for 30 s (21).

All procedures were approved by the Ethic Committee for Animal Experiments from the University of Zaragoza. The care and use of animals were performed accordingly with the Spanish Policy for Animal Protection RD53/2013, which meets the European Union Directive 2010/63 on the protection of animals used for experimental and other scientific purposes.

### Sample Collection

Blood was taken from 6 hemizygous SOD1G93A males and females and their wildtype (WT) littermates at 40 days of age (P40), which corresponds to the asymptomatic stage of the disease. On the other hand, serial plasma samples were taken from 84 SOD1G93A mice at early symptomatic (60 days of age) (P60), symptomatic (90 days of age) (P90), and terminal or endpoint stages (from 112 to 150 days of age). Plasma samples at P60 and P90 were obtained from submandibular bleeding, whilst those at terminal stage were collected from blood extracted by cardiac puncture. For this, the mice were euthanized with CO_2_, then blood was extracted and transferred to EDTA coated mini vacutainer tubes (BD Biosciences, USA). Then, blood was centrifuged at 3,000 rpm for 10 min at 4°C within 30 min from extraction, plasma was collected and immediately frozen in dry ice and stored at −80°C.

### Cytokine Array Assay

A cytokine array assay was performed to analyze protein levels of 97 cytokines (Mouse Cytokine Antibody Array G6, Raybiotech, Inc.) in plasma samples from 6 WT and 6 SOD1G93A mice at P40. The array was carried out by the service offered by Raybiotech, Inc.

### Multiplex and Quantibody Immunoassays

From the 84 serial plasma samples obtained, 32 SOD1G93A mice showing the longest (from 133 to 150 days old) and the shortest (from 112 to 126 days old) survival time were selected to study cytokine levels during disease progression through a multiplex and quantibody immunoassays. The multiplex immunoassay (ProcartaPlex Multiplex Immunoassay, Affymetrix eBioscience, Thermo Fisher Scientific Inc.) was carried out for the protein analysis of eotaxin-1, IL-2, IL-6, IL-10, IL-13, macrophage inflammatory protein (MIP)-1 alpha (α), and receptor activator of nuclear factor kappa-B ligand (RANKL) in plasma from 20 SOD1G93A mice. Additionally, a total of 9 cytokines were analyzed using a quantibody array (Quantibody® Mouse Cytokine Array, Raybiotech, Inc.) in samples from 12 SOD1G93A mice. The cytokines studied were: chemokine (C-C motif) ligand 21 (CCL21 or 6Ckine), activin receptor-like kinase (ALK)-1, tumor necrosis factor (TNF) ligand superfamily member 8 (TNFSF8 or CD30 L), Galectin-1, TNF receptor superfamily member 18 (TNFRSF18 or GITR), interleukin 17B receptor (IL-17B R), MIP-3 beta (β), TNF receptor superfamily member 19 (TNFRSF19 or TROY), and vascular endothelial growth factor (VEGF)-D.

### Statistical Analysis

Comparisons of results obtained in cytokine array assay between the WT and SOD1G93A groups were made using *t*-tests or Mann-Whitney U-tests, according to data distribution analyzed by Shapiro-Wilk test. For cytokines whose expression was not quantifiable at some point of the disease progression, relationships between positive or null cytokine expression and survival time were evaluated through a Kaplan-Meier analysis. On the other hand, comparisons of means of quantifiable cytokines between short-and long-lived mice were analyzed by unrelated *t*-test or Mann-Whitney U-test. Finally, correlations between cytokines levels and survival time were assessed using Pearson's correlation analysis.

Statistical analysis was performed using SPSS (version 20, IBM, Armonk, NY) and GraphPad Prism Software (version 5, La Jolla, CA). All of the values were expressed as the mean ± standard error of the mean (SEM). Significance levels were set at a *p* < 0.05.

## Results

### Sixteen Cytokines Were Dysregulated at the Asymptomatic Stage in Transgenic SOD1G93A Mice

**Figure 1:**
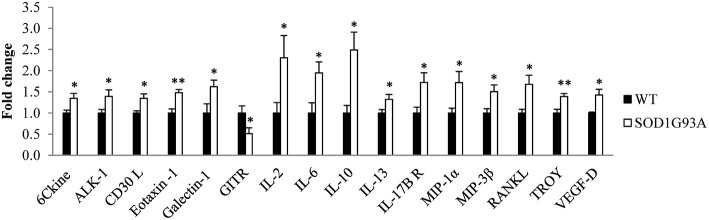
Dysregulation of 16 cytokines in plasma from transgenic SOD1G93A mice at the asymptomatic stage of the disease (P40). A total of 97 cytokines were analyzed through a cytokine array assay to evaluate their levels in plasma from six wildtype (WT) and six SOD1G93A mice at P40. T-student and Mann-Whitney U-tests were used. 16 cytokines (6Ckine, ALK-1, CD30 L, eotaxin-1, galectin-1, GITR, IL-2, IL-6, IL-10, IL-13, IL-17B R, MIP-1α, MIP-3β, RANKL, TROY, and VEGF-D) were found dysregulated in transgenic SOD1G93A mice. Bars represent mean ± standard error of the mean (SEM). *p < 0.05, **p < 0.01.

Plasma was analyzed from 6 hemizygous SOD1G93A males and females and their wildtype littermates at P40, the asymptomatic stage, through a cytokine array assay. The analysis identified 15 cytokines significantly upregulated (6Ckine, ALK-1, CD30 L, galectin-1, eotaxin-1, IL-2, IL-6, IL-10, IL-13, IL-17B R, MIP-1α, MIP-3β, RANKL, TROY, and VEGF-D) and one significantly downregulated (GITR) in SOD1G93A mice (Figure 1). Therefore, our next step was to investigate the inflammatory response to the disease progression through the analysis of these 16 cytokines in serial plasma samples.

### Expression Profile of ALK-1, Eotaxin-1, Galectin-1, GITR, and IL-17B R Along Disease Progression Was Associated With Shorter Survival Rate of Transgenic SOD1G93A Mice

Firstly, multiplex and quantibody immunoassays were performed in serial plasma samples from the mutant animals to investigate how levels of the cytokines altered in the asymptomatic stage evolved along disease progression. Three plasma samples were obtained from each animal, corresponding to early symptomatic (P60), symptomatic (P90) and terminal stage (endpoint). Then, the 32 animals showing the shortest and the longest survival time were selected to assess the effect of cytokine expression on longevity at each stage of the disease.

Expression of 7 cytokines (6Ckine, ALK-1, CD30 L, GITR, IL-17B R, MIP-3β, and VEGF-D) was not quantifiable in all stages. Consequently, two animal groups were formed depending on the positive or null expression of these cytokines in plasma. Then, Kaplan-Meier analysis was used to evaluate relationships between longevity and presence/absence of cytokine expression. This analysis revealed that positive expression of ALK-1, GITR at P60 and P90, and IL-17B R at P60 was associated with shorter survival rate (Table 1). In contrast, no associations were found between expression and longevity in the terminal stage. This means that the animals having a longer survival rate had undetectable levels of these cytokines in symptomatic stages of the disease.

**Table 1:** Relationships between positive or null expression of cytokines with survival time in transgenic SOD1G93A mice at each stage of the disease.

**Cytokines**	**P60**	**P90**	**Endpoint**
6Ckine	No sig	No sig	No sig
ALK-1	^*^p = 0.016	^**^p = 0.003	No sig
CD30 L	No sig	No sig	No sig
GITR	^**^p = 0.009	^*^p = 0.019	No sig
IL-17B R	^*^p = 0.016	No sig	No sig
MIP-3β	No sig	No sig	No sig
VEGF-D	No sig	No sig	No sig

*Twelve animals were grouped depending on the presence or absence of cytokines measured in their plasma. The relationships between their expression and their survival time were evaluated through a Kaplan-Meier analysis and Log-Rank test. Positive expression of ALK-1, GITR, and IL-17B R at P60, and ALK-1 and GITR at P90 was found in mice with shorter survival*.

* *p < 0.05*,

** *p < 0.01*.

**Figure 2:**
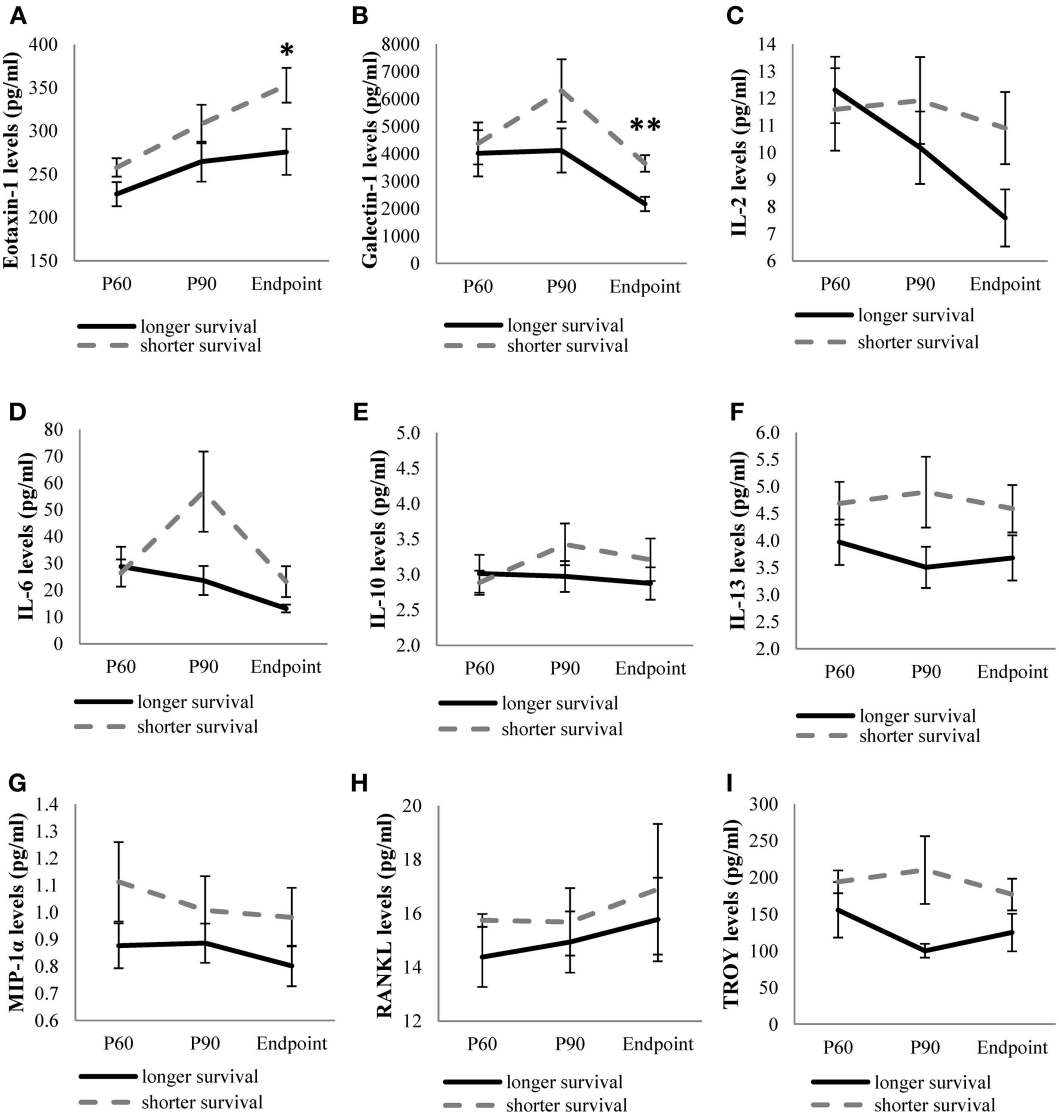
Levels of quantifiable cytokines along disease in transgenic SOD1G93A mice depending on their survival time. Cytokine levels of eotaxin-1 **(A)**, galectin-1 **(B)**, IL-2 **(C)**, IL-6 **(D)**, IL-10 **(E)**, IL-13 **(F)**, MIP-1α **(G)**, RANKL **(H)**, TROY **(I)** obtained from mice with the shortest survival rate (112–126 days old) were compared with those obtained from mice with the longest survival rate (133–150 days old) at each stage of the disease (P60, P90, and endpoint). The sample size was 20 mice for eotaxin-1, IL-2, IL-6, IL-10, IL-13, MIP-1α, RANKL, and 12 mice for galectin-1 and TROY. Unrelated *t*-test or Mann-Whitney U-tests were used. Eotaxin-1 and galectin-1 levels were significantly increased at terminal stage in SOD1G93A mice with the shortest survival rate. Graphics show mean ± standard error of the mean (SEM). *p < 0.05, **p < 0.01.

On the other hand, levels of the remaining cytokines (eotaxin-1, galectin-1, IL-2, IL-6, IL-10, IL-13, MIP-1α, RANKL, and TROY) were continuously monitored in the three stages studied. We observed higher expression of the cytokines in animals showing short survival rate, although only eotaxin-1 and galectin-1 were significantly upregulated at endpoint in animals exhibiting shorter survival compared to the levels observed in long-lived animals (Figure 2).

### Eotaxin-1, Galectin-1, IL-2, IL-6, MIP-1α, and TROY Levels at P90 or Endpoint Negatively Correlated With Survival Time

To analyze more in depth whether the levels of quantifiable cytokines (eotaxin-1, galectin-1, IL-2, IL-6, MIP-1α, and TROY) could influence longevity of mice, the expression levels of cytokines were correlated with the survival rate in SOD1G93A mice. Pearson correlation analysis showed negative correlations between survival rate of the animals and the levels of eotaxin-1, galectin-1, IL-2, IL-6, MIP-1α and TROY at P90 or endpoint (Table 2), revealing that the higher the expression of these cytokines the lower the survival rate in SOD1G93A mice.

**Table 2:** Correlations between cytokine expression and survival time at each stage.

**Cytokines**	**Stage**	***p*-value**	**Pearson**
Eotaxin-1	Endpoint	^*^0.046	−0.450
Galectin-1	Endpoint	^**^0.009	−0.744
IL-2	Endpoint	^*^0.032	−0.481
IL-6	P90	^*^0.045	−0.465
	Endpoint	^*^0.024	−0.544
MIP-1α	Endpoint	^*^0.048	−0.448
TROY	P90	^*^0.042	−0.620

*Relationships between quantifiable cytokines levels and survival time at P60, P90, and endpoint were assessed using Pearson's correlation analysis. The sample size was 20 mice for eotaxin-1, IL-2, IL-6, MIP-1α, and 12 mice for galectin-1 and TROY. Negative correlations were found between survival time and levels of IL-6 and TROY at P90, and eotaxin-1, galectin-1, IL-2, IL-6, and MIP-1α at endpoint*.

* *p < 0.05*,

** *p < 0.01*.

## Discussion

The active role of pro-inflammatory cytokines, enhancing the development of ALS, has been reported in both animal models and ALS patients (7, 13, 17, 22). The possibility of studying the interplay between inflammation and disease progression in easily accessible tissues, such as plasma, is an essential step to identify potential biomarkers that could help for an early prognosis of the disease. An easily testable panel of potential predictors of disease progression could help to validate the findings from the animal models to ALS patients and, thus, facilitate the translation of these at clinical level. In this sense, we have studied a panel of 97 cytokines, some of them newly related to ALS in this study, in serial plasma samples from transgenic SOD1G93A mice. The possibility of analyzing these cytokines in the asymptomatic stage in this animal model can provide information about early factors involved in disease progression and in survival, normally not possible to obtain in ALS patients, validating in this way their prognostic capacity.

Firstly, we performed a cytokine array assay to measure expression levels of 97 cytokines in plasma from WT and transgenic SOD1G93A mice at the asymptomatic stage of the disease. Among all cytokines, 16 of them were found significantly dysregulated in transgenic SOD1G93A mice. These results support the fact that immune system was already altered in the asymptomatic stage of the disease through a dysregulation of both pro- and anti-inflammatory cytokines, as previously reported (17, 18, 23). Moreover, these findings reveal for the first time the role of some cytokines that had not been related to ALS yet, including chemokines belonging to the tumor necrosis factor (TNF) and tumor necrosis factor receptor (TNFR) superfamilies (CD30 L, GITR, TRANCE, TROY), chemokine ligands family (6Ckine, MIP-3β) and other cytokines (ALK-1, VEGF-D). These premature alterations may have a prejudicial role in exacerbating pathologic mechanisms in ALS.

Considering that a compensatory response to inflammation can promote longer survival, as previously reported in this animal model (24, 25), our next step was to analyze more in depth the potential modulatory effect of these cytokines on the longevity of the animals. In this study, the differences in the survival rate of SOD1G93A mice let us monitor the expression of the studied cytokines along disease progression in two groups of animals, short- and long-lived animals. In addition, the expression levels of some of the selected cytokines could not be continuously monitored along the disease progression in the SOD1G93A mice. Therefore, we classified cytokines in two groups, those that were quantifiable along the disease progression, and those that could not been continuously quantified. Regarding this last group (6Ckine, ALK-1, CD30 L, GITR, IL-17B R, MIP-3β, and VEGF-D), the association between the positive or null expression of these cytokines and the survival rate of SOD1G93A mice was analyzed. We observed that the positive expression of ALK-1, GITR and IL-17B R at symptomatic stages (P60 and P90) was associated with shorter survival rate. ALK-1 is a type I cell surface receptor for the transforming growth factor-β (TGF-β) family of proteins involved in endothelial cells biology and angiogenesis (26). GITR (TNFSF member 18) plays an important role in expansion of peripheral regulatory T cells (Tregs) (27, 28), which have been associated with slow progression of ALS (29, 30). However, GITR can be also expressed on effector T cells, favoring inflammatory process that could accelerate the neurodegenerative progression in a Ying-Yang unbalance, which is more in accordance with our findings that showed an association of GITR expression at the symptomatic stages in transgenic animals with shorter survival rates (31). Regarding IL-17B R, it is part of the complex of chains for the IL-17E receptor together with IL-17A R. Although no involvement of IL-17B R in ALS has been reported so far, levels of IL-17A were found elevated in serum of ALS patients compared to control subjects (32). In addition, IL-17E promotes Th2 immune response (33), releasing anti-inflammatory cytokines, which could counteract the inflammation and therefore, slowing down disease progression.

In relation to the cytokines whose expression was quantifiable in all stages (eotaxin-1, galectin-1, IL-2, IL-6, IL-10, IL-13, MIP-1α, RANKL, and TROY), our findings showed that eotaxin-1 and galectin-1 levels were significantly upregulated at the endpoint stage in the transgenic mice that exhibited shorter survival rate. These results suggest that increased levels of these cytokines may aggravate the disease course, especially at terminal stages. Eotaxin-1 is a potent eosinophil chemoattractant whose role in neurodegenerative diseases such as Alzheimer's disease (AD), Huntington's disease, multiple sclerosis and ALS has been previously studied (34, 35). In accordance with our results, increased levels of eotaxin-1 in serum and in cerebrospinal fluid (CSF) from ALS patients vs. controls were observed in previous studies (36, 37). In contrast, increased eotaxin-1 levels in CSF from ALS patients correlated with slow disease progression, suggesting that it might play a protective role in ALS (38). These differences could be due to the different nature of the studied tissue in which eotaxin-1 was measured. In fact, CSF and plasma cytokines do not always show similar values, as previously reported (39–41). Galectin-1 is a member of the β-galactoside-binding lectin family which has been associated with activated astrocytes and neurofilamentous lesions in the spinal cord of SOD1G93A mice and ALS patients before the onset of symptoms (42, 43). In contrast, it has been reported that galectin-1 can protect against neurodegeneration through glycosylation-dependent inactivation of M1 cells (microglia inducing neurotoxic T-cell response) (44) and that oxidized galectin-1 shows neuroprotective effect by promoting axonal regeneration (45, 46). Therefore, galectin-1 seems to have a dual role depending on its oxidized form or the disease stage. A possible explanation for this might be that galectin-1 could not be able to exert its protective effect due to underlying mechanisms, for instance, when there is a wrong addressing to the target tissues or an inactivation of its action through other effectors. Therefore, it would be interesting to study galectin-1 expression simultaneously in plasma and target tissues to elucidate its role in ALS more accurately.

Considering the fact that decreased levels of some of these cytokines were associated with a longer lifespan in transgenic mice, we analyzed the relationship of the quantifiable cytokines along disease progression in transgenic SOD1G93A mice with their survival rate. Pearson analysis showed that IL-6 and TROY at the symptomatic stage, and eotaxin-1, galectin-1, IL-2, IL-6, and MIP-1α at terminal stage were negatively correlated with survival time of mice. These findings reflected that the inflammatory response in the animals from the symptomatic stage could favor faster disease progression. Regarding eotaxin-1 and galectin-1, their levels were increased in animals which lived shorter. However, opposing results have been found in the literature, as aforementioned, suggesting that more research needs to be undertaken to clarify their participation in ALS progression. On the other hand, increased levels of IL-2 in plasma correlated with poor survival rate, which is consistent with other studies (16, 47). On the contrary, no correlation was found with disease duration in neither CSF nor serum of ALS patients in other works (48). IL-2 could exert a dual role in ALS by induction of Tregs and by activation of natural killer (NK) cells, which are cytotoxic for some neurons (16, 49). One possible explanation could be based on the activation of NK cells by higher IL-2 levels at terminal stage, contributing to faster disease progression. IL-6 has been related to ALS in several studies (11, 13, 16, 50–52), although alterations of this cytokine do not seem to be specific to ALS. Particularly, it has been linked to other neurodegenerative diseases, such as AD and Parkinson's disease (53, 54). In our study we observed that higher IL-6 levels at P90 and endpoint correlated with shorter lifespan. Increasing IL-6 levels have been described along disease progression in plasma from ALS patients, although no correlation with the lifespan was found (16, 48). On the contrary, reducing IL-6 levels along time have also been reported in ALS patients (11). These opposing results could suggest that the disease stage when IL-6 levels were measured could be relevant to promote a favorable or detrimental effect in the transgenic animal. In addition, the dual role of IL-6 as a pro- and anti-inflammatory interleukin can also depend on where this cytokine is secreted, showing an anti-inflammatory effect in the skeletal muscle (18, 55). MIP-1α is a neutrophil chemoattractant and activator (56), and it is expressed by astrocytes in CNS in response to inflammation (57). We found an association of increased levels of MIP-1α at endpoint with shorter survival rate. Accordingly, increased MIP-1α levels have been reported in both CSF and serum samples from ALS patients and they correlated negatively with the disease progression (58). In contrast, other studies suggested a positive correlation with the disease progression (48, 58), while some others did not find any variation in this chemokine in ALS patients respect to healthy controls (37), revealing again the controversy of results found in the literature regarding these chemokines. TROY levels negatively correlated with lifespan at the symptomatic stage of the disease. Very little is found in the literature on TROY's involvement in ALS. TROY is a TNF receptor family member expressed in the adult nervous system and forms a functional receptor complex with RTN4R (NgR1) and LINGO-1 involved in mediating myelin inhibition (59, 60). In addition, TROY is a negative regulator of oligodendrocyte development (61). Alterations in genes involved in myelin structure and function, and affected oligodendrocytes in the spinal cord have been described in a mouse model, contributing to the disruption of axonal integrity and motor neuronal death (62, 63). Since TROY is involved in both regulation of myelination and oligodendrocyte development, our findings could suggest that TROY could be exacerbating disease course, exerting its major effect at the symptomatic stage.

**Figure 3:**
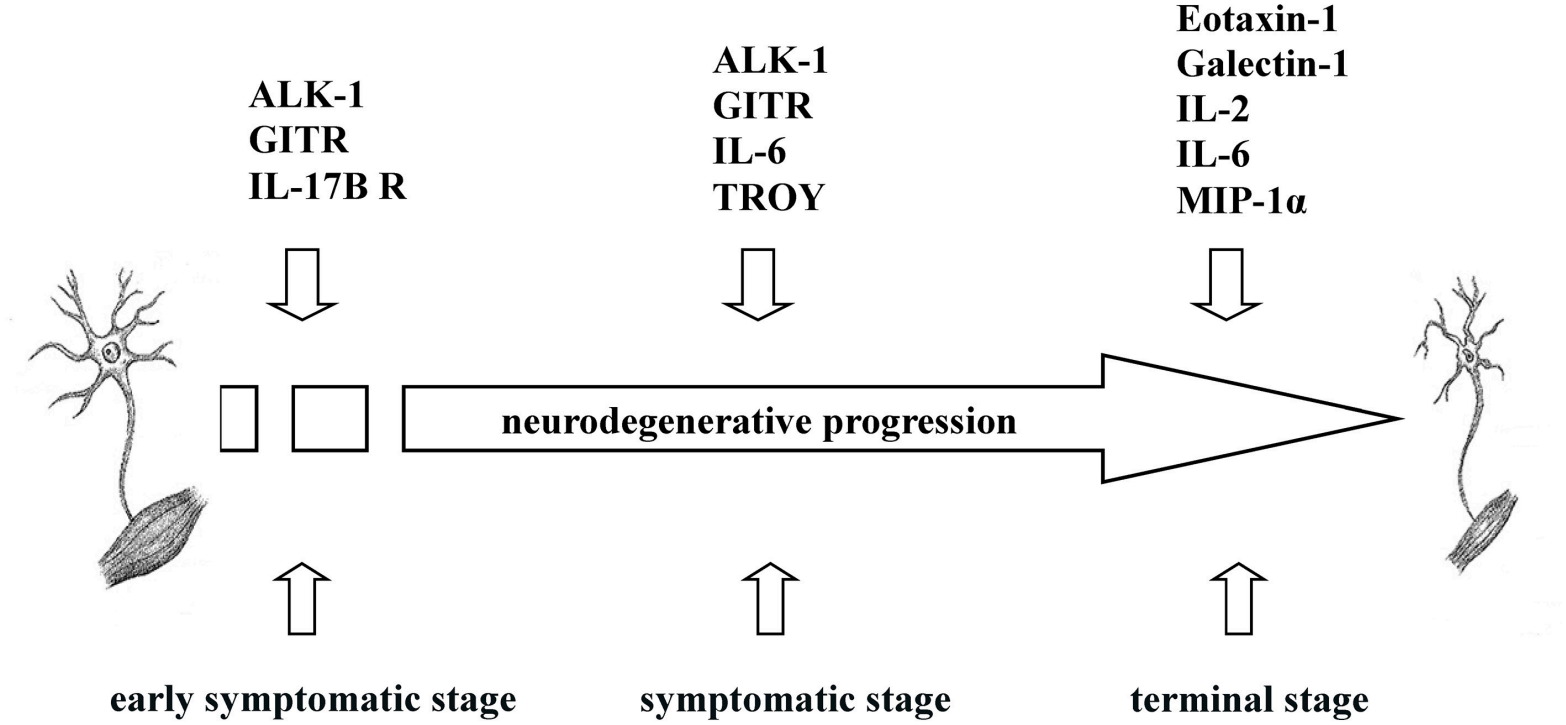
Overview of the involvement of ALK-1, eotaxin-1, galectin-1, GITR, IL-2, IL-6, IL-17B R, MIP-1α, and TROY in ALS. Based on the findings obtained, ALK-1, eotaxin-1, galectin-1, GITR, IL-2, IL-6, IL-17B R, MIP-1α, and TROY exerted different inflammatory roles in transgenic SOD1G93A mice depending on the disease stage, influencing their survival rate.

To sum up, we found that increased levels of five cytokines (ALK-1, eotaxin-1, galectin-1, GITR, IL-17B R) were associated with transgenic mice that exhibited a shorter survival rate at specific time points during disease progression, which may indicate that the pro-inflammatory nature of these cytokines could influence the survival time in transgenic mice. Similarly, negative correlations between the survival rate and the expression of six cytokines (eotaxin-1, galectin-1, IL-2, IL-6, MIP-1α, and TROY) at P90 or terminal stages were found. Therefore, pro-inflammatory cytokines secreted in plasma could modulate peripheral inflammatory response depending on the stage of the disease at which they are released; consequently, high levels of these cytokines could aggravate the course of the disease favoring shorter longevity in the animals (Figure 3).

Taken together, our results showed a panel of cytokines, some of them first described in ALS, in serial plasma samples that influenced survival in transgenic SOD1G93A mice, highlighting an activation of immune system dependent on the disease stage. However, to date there is not a consensus for the function of any cytokine in ALS, as their expression in blood seems to be very variable. The potential advantages of finding a biomarker in blood are minimal invasiveness and easy accessibility; however, maybe it is not the best option for measuring and studying cytokines as prognostic biomarkers, as those molecules are susceptible to change by other alterations occurring in the subject. Nevertheless, the findings obtained in this study confirmed that a predominant pro-inflammatory status could exacerbate the disease course; therefore, cytokines could be considered molecular targets for potential therapies.

## Ethics Statement

All procedures were approved by the Ethic Committee for Animal Experiments from the University of Zaragoza. The care and use of animals were performed accordingly with the Spanish Policy for Animal Protection RD53/2013, which meets the European Union Directive 2010/63 on the protection of animals used for experimental and other scientific purposes.

## Author Contributions

AC, AG-R, PZ, and RO were implied in the experimental design. LM-M and MdlT collected plasma samples from mice at different stages and performed immunoassays to analyze cytokine levels. AC and LM-M interpreted the data and performed the statistical analysis. AC, JT, LM-M, and RO were major contributors in writing the manuscript. All authors read and approved the final manuscript.

### Conflict of Interest Statement

The authors declare that the research was conducted in the absence of any commercial or financial relationships that could be construed as a potential conflict of interest.

## Abbreviations

6Ckine: Chemokine (C-C motif) ligand 21
AD: Alzheimer's disease
ALK-1: Activin receptor-like kinase 1
ALS: Amyotrophic lateral sclerosis
C9orf72: Chromosome 9 Open Reading Frame 72
CCL21: Chemokine (C-C motif) ligand 21
CD30 L: Cluster of Differentiation 30 ligand
CSF: Cerebrospinal fluid
DNA: Deoxyribonucleic acid
EDTA: Ethylenediaminetetra-acetic acid
FUS: Fused in sarcoma
GITR: Glucocorticoid-induced tumor necrosis factor receptor
IL-2: Interleukin 2
IL-6: Interleukin 6
IL-10: Interleukin 10
IL-13: Interleukin 13
IL-17A R: Interleukin 17A receptor
IL-17B R: Interleukin 17B receptor
LINGO-1: Leucine rich repeat and Immunoglobulin-like domain-containing protein 1
MIP-1α: Macrophage inflammatory protein 1 alpha
MIP-3β: Macrophage inflammatory protein 3 beta
NgR1: Neuregulin 1
NK: Natural killer
P40: Postnatal day 40
P60: Postnatal day 60
P90: Postnatal day 90
PCR: Polymerase chain reaction
RANKL: Receptor activator of nuclear factor kappa-B ligand
RNA: Ribonucleic acid
RTN4R: Reticulon 4 Receptor
SEM: Standard error of the mean
SOD1G93A, Superoxide dismutase 1: with glycine to alanine transition at position 93
SPSS: Statistical Package for the Social Sciences
TARDBP: Tar DNA-binding protein gene
Th2: T Helper Cell Type 2
TLS: Translocation in liposarcoma
TNF: Tumor necrosis factor
TNFR: Tumor necrosis factor receptor
TNFRSF18: Tumor necrosis factor receptor superfamily member 18
TNFRSF19: Tumor necrosis factor receptor superfamily member 19
TNFSF: Tumor necrosis factor ligand superfamily
TNFSF8: Tumor necrosis factor ligand superfamily member 8
Treg: T regulatory cells
TROY: Tumor necrosis factor receptor superfamily member 19
VEGF-D: Vascular endothelial growth factor D
WT: Wildtype.
